# Outcomes of preoperative medical consultations for elective non cardiac surgical procedures at tertiary care center

**DOI:** 10.12669/pjms.42.1.11910

**Published:** 2026-01

**Authors:** Fahad Tariq, Faraz Shafiq

**Affiliations:** 1 Fahad Tariq Senior Registrar, Department of Anesthesiology, Tabba Heart Institute, Karachi, Pakistan; 2 Faraz Shafiq Associate Professor, Department of Anesthesiology, Aga Khan University Hospital, Karachi, Pakistan

**Keywords:** Tertiary care centers, Elective surgical procedures, Consultation

## Abstract

**Objective:**

To evaluate the outcome of medical consultations requested at the time of pre-operative anaesthesia assessment.

**Methodology:**

After taking exemption from ethical review committee, this descriptive cross-sectional study was conducted for one year at our tertiary care center. All adult patients scheduled for elective surgeries required consultation from anaesthesia physicians were included. Relevant clinical data was recorded prospectively on predesigned Performa. Outcomes were categorized as risk stratification, optimizing medical management or postponement of surgical procedure.

**Results:**

Among 264 patients (mean age 59.9 years, predominantly male, ASA III), hypertension, diabetes, and IHD were the common comorbid conditions. About 47% of these consultations were requested for intermediate risk procedures, while cardiology opinion was requested in (63%) of cases. Based on these consultations, 87.5% of patients received risk prediction, medical management was advised in 64% and in only 7.2% of patients the planned surgeries were postponed. Comparison between variables showed Myasthenia Gravis (50%), Pulmonary embolism (33%) and IHD (30%) were the common reasons of falling into high-risk group. Most patients with Asthma (69%), IHD (68%) and Hypothyroidism (52%) required medical intervention preoperatively (p = 0.496). IHD was mainly responsible for the reason of delaying surgery (13.6%). Similarly, 69% of cardiac consultations ended up with case postponement, and 53.5% of patients were advised with medical management (p = 0.001).

**Conclusion:**

The substantial disease burden and proposed risk stratification including recommendations regarding medical optimization justified the need of preoperative consultations in our patient populations. However, their impact on improving postoperative outcomes requires further evaluation.

## INTRODUCTION

Preoperative anaesthesia evaluation is a routine, but essential component of perioperative path of patients planned for surgical intervention.^1^ The purpose of this assessment is mainly to evaluate the base line health status, and identification of comorbid conditions. Based on this, optimization and risk stratification can be predicted. Moreover, this assessment is also fundamental, for preoperative shared decision making and related planning. Multidisciplinary team input is helpful to mitigate the risk associated with base line health condition and surgical intervention.

Formal pre-operative consultations are routinely sought to facilitate the input of medical specialists and internists.^2^ The process is defined as the formal way of assessing patient before the planned surgery.^3^ The opinions are essential not only to highlight base line comorbid conditions, but also to improve associated outcomes. Thereby, helpful to declare individualize care plan suitable for particular surgery.^4^ Commonly encountered comorbid conditions in surgical population are Diabetes mellitus (DM), Hypertension (HTN), Ischemic Heart Disease (IHD) and Chronic obstructive pulmonary disease (COPD).^5^ The impact of them is substantial, with estimation that out of all the patients who undergo elective surgery, 15-20% develop serious complications and about 0.5-2% ends up with adverse outcomes.^6^

The paradigm of multidisciplinary care emphasizes comprehensive patient workup to optimize these conditions prior to surgery.^7^ However, utilization preoperative consultation needs critical evaluation in terms of consumption of resources including straining manpower, laboratory, and delay in planned procedure. Ultimately, this would add up to the health care related cost.^8^ Conversely, literature also demonstrates beneficial aspects in terms of improving recovery profiles, reducing hospital length of stay and perioperative complications in surgical population.^9^ Considering the overall disease burden and baseline health status of our population, it is essential to evaluate the effectiveness of local practices. Hence, the need for these consultations and their effectiveness require consideration. This study was aimed to evaluate the effectiveness of these consultations. The objective of the study was to evaluate the outcome of medical consultations requested at the time of preoperative anaesthesia assessment at our tertiary care center. The outcomes planned to be measured were predicting risk stratification, recommending medical management or postponement of surgical procedure.

## METHODOLOGY

A descriptive cross-sectional study was conducted at our tertiary care unit for a period of one year from January - December 2022.

### Ethical Approval:

The study protocol was reviewed and granted exemption Chairperson of Ethics Review Committee (ERC) of The Aga Khan University, Karachi (Reference number: 2020-5597-15244; Date: 28-Dec-2020).

All adult patients planned for elective non cardiac surgical procedures, and for whom preoperative consultation was generated by anaesthesia physicians were enrolled in study protocol. Patients, who either left against medical advice or lost their follow-up were excluded. The sampling technique was consecutive. Sample size calculation was based on previous study by Dogan et al. They reported 85 cancellations, out of 1285 planned cases. Based on it, total 264 patients were required to estimate expected outcome (Surgery Cancelled = 6.6% (85/1285) with 3% margin of error at 95% confidence interval.^10^

Preoperative anaesthesia assessments of all patients were carried out by trained anaesthetists. This was carried out either at pre-operative clinic or in surgical wards, under direct or remote supervision of a consultant anaesthetist. After thorough evaluation, those who required additional consultation were included in study protocol. The medical record numbers of selected patient were retrieved using health information management system. One of the primary investigators was responsible for all the data collection. The relevant demographic and preoperative data were recorded on a predesigned Performa. The measured outcomes were followed up by consultation process. This includes risk stratification, optimization in terms of medical management, or postponement of planned surgical procedure.

### Statistical Analysis:

Data was analyzed using Statistical Package for Social Science (SS-19). Mean and standard deviation was estimated for age, weight, height, and BMI. The frequency and percentages were calculated for measures including gender, ASA physical status, comorbid conditions, type of surgical procedures and frequency of consultations. Histogram and Kolmogorov-Smimov were used to check the normality of quantitative data. For non-normal (heterogeneous) data, median and IOR were reported. Effect modifiers were controlled by stratification, and the effect of these modifiers was observed on the outcome by using chi-square or Fisher exact test. P value of less than 0.05 was considered significant.

## RESULTS

**Fig.1:**
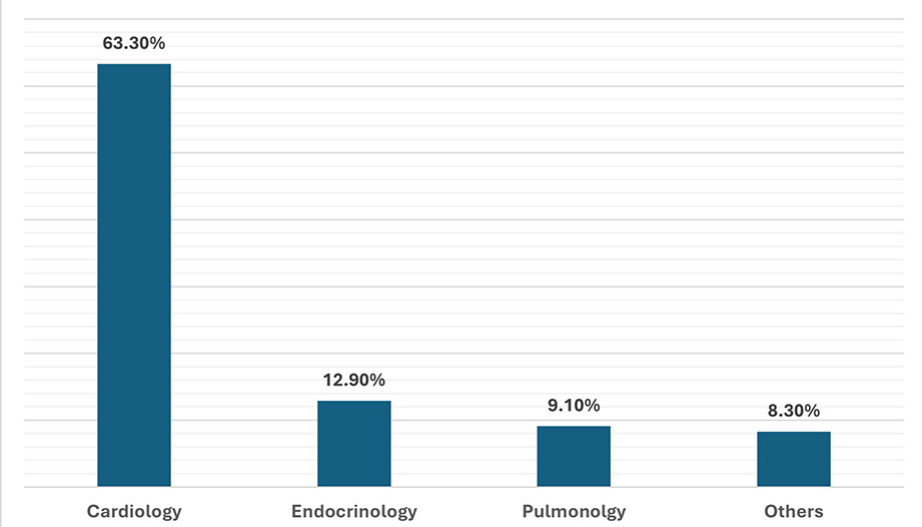
Specialty wise consultations.

After fulfilling the eligibility criteria, a total of 264 participants were enrolled in the study protocol. There were no dropouts and none of selected patient lost the follow-up. The demographic characteristics of patients are mentioned in Table-I. This shows 63% of patients had associated comorbid conditions and more than half of them (62.5%) were scored with ASA physical status of grade-III. Review of comorbid conditions revealed the predominance of HTN (76%), DM (58%) and IHD (25%). For majority of them (63%) cardiology opinion was requested, followed by endocrinology 13%, only 9% of cases pulmonology input was required Fig.1. Categorization of surgical risk revealed 29% of them belongs to high-risk surgical procedures, 34% from intermediate-risk and 47% were related to low-risk surgical procedures.

**Table-I:** Participant Demographics, Comorbidities, and Analysis Outcomes.

Variables	(n = 264)
Age Mean (SD)	59.9 (15.0)
BMI Mean (SD)	27.1 (5.25)
***Gender***	
Female	45.8%
Male	54.2%
***ASA Physical Status***	
I	0.4%
II	29.9%
III	62.5%
IV	7.2%
***Comorbid Conditions***	
HTN	76.1%
DM	58%
IHD	25%
Hypothyroidism	8.7%
Asthma	4.9%
Epilepsy	4.2%
Others	18.7%
***Surgical Procedures***	
General Surgery	25.4%
Urology	22.0%
Orthopedics	15.9%
Gynecology	12.5%
Neurosurgery	9.1%
Others	15.3%
***Risk Categorization***	
Low risk procedures	47%
Intermediate risk procedures	34%
High risk procedures	11%

**Fig.2:**
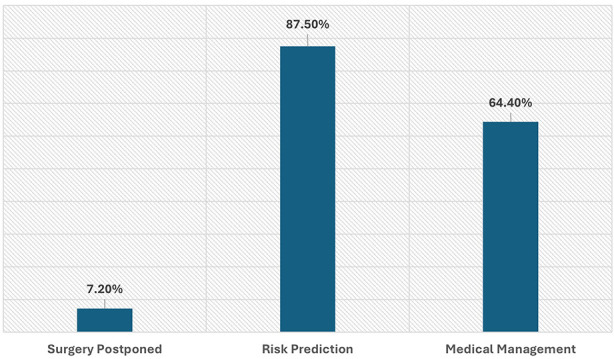
Outcome of Preoperative Consultations.

The outcome of these consultations revealed that, in only 7.2% (n=19) surgical procedure was postponed. Most of these patients received recommendations in terms of risk prediction, which was determined in 231 patients (88%). The risk stratification was categorized as low (16.3%), intermediate (57%), or high risk (14%). Preoperative optimization is an important aspect of generating expert opinion and hence such consultations. Out of 264 patients, 64.4% patients underwent some medical management preoperatively (Fig.2).

**Fig.3:**
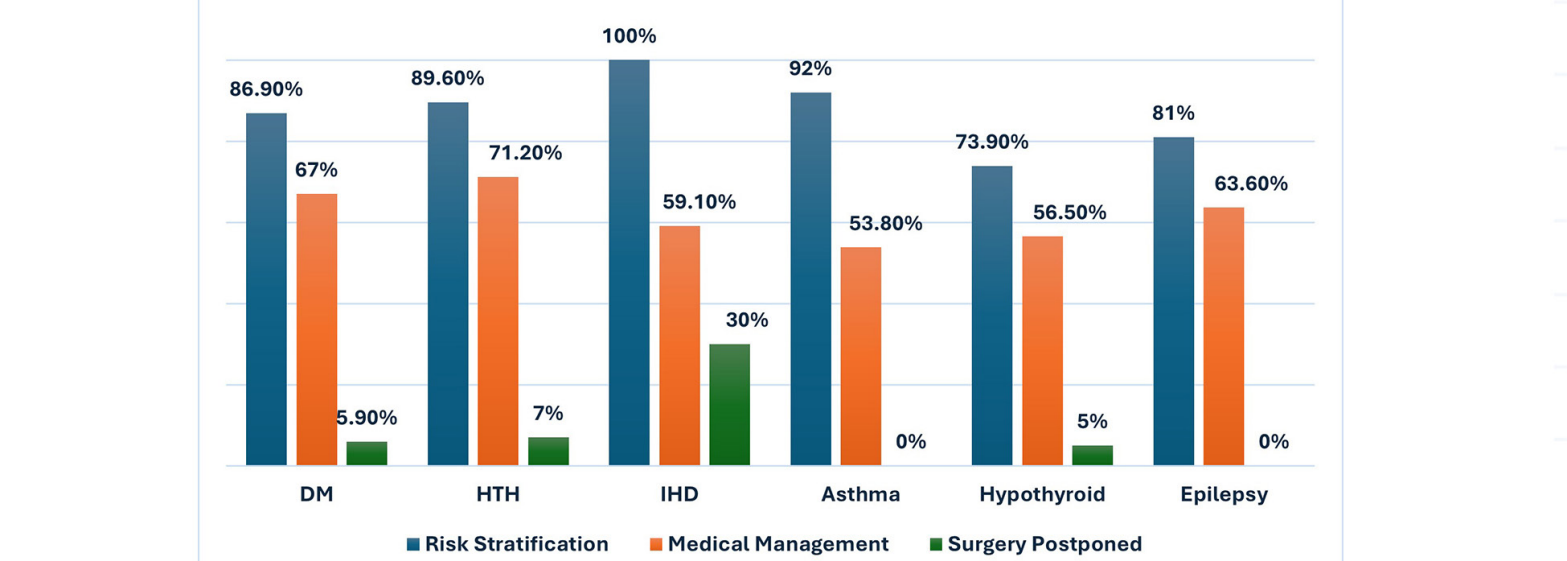
Association of comorbid conditions with measured outcomes.

Cross comparison between various variables showed more than half of these consultations were generated in patients having HTN (68.7%) and DM (68.6%). Most patients with HTN (67.2%), DM (66%), Asthma (58.3%), Epilepsy (66.7%) and hypothyroidism (58.8%) fell into intermediate risk category. Preoperative medical conditions like Myasthenia Gravis (50%), Pulmonary embolism (33%) and IHD (30%) were the common reasons high-risk group with p-value <0.001. IHD was mainly responsible for the reason of delaying surgery. Overall, 30% of patients with IHD were not proceeded with the surgery (p = 0.041). Similarly, most patients with Asthma (69%), IHD (68%) and Hypothyroidism (52%) required medical intervention preoperatively for preoperative optimization (p = 0.496) Fig.3. Overall, 69% cardiac consultations ended up with case postponement and in 53.5% were advised with medical management (p = 0.001).

**Fig.4:**
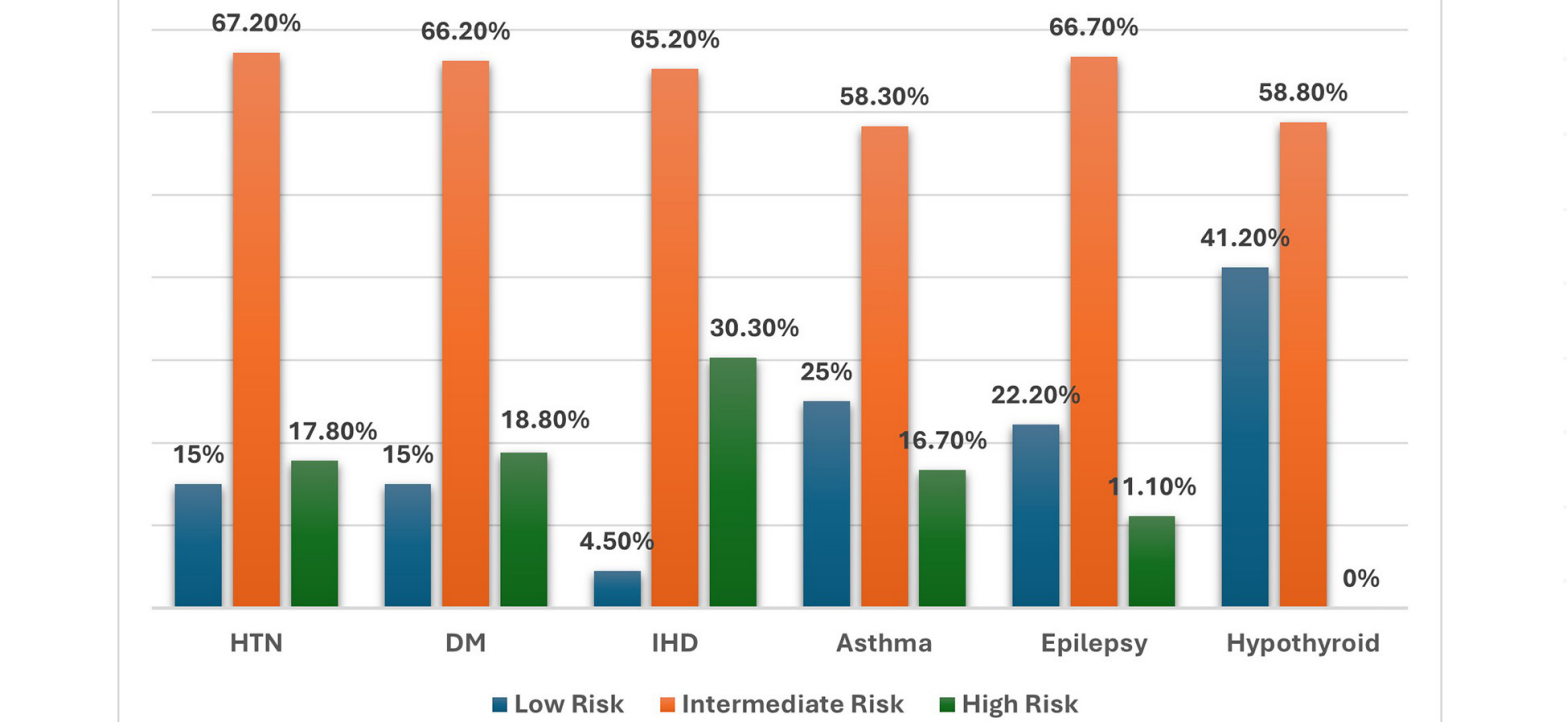
Association of comorbid conditions with risk stratification.

## DISCUSSION

Pre-operative medical consultation is pivotal for optimizing a patient’s health status and facilitating comprehensive surgical planning. The necessity and nature of these consultations are influenced by variations in patient demographics and disease pattern. This study underscores the utility of additional consultations in terms of preoperative optimization specifically in our healthcare context. Where, the issues of compliance, adherence to medical advice and infrequent follow-ups are prevalent. These issues are often compounded by socioeconomic, educational and administrative factors.^11^

Our findings demonstrated the significant association of preoperative consultation with comorbid conditions like HTN, DM, IHD and Asthma. Interestingly, many of them were poorly controlled and advised medical management accordingly. Similarly, 57% of patients were labeled as intermediate risk, which highlighted the underlying issues of management and compliance. However, accessing the overall utility of these opinions in terms of outcomes is challenging. Beckerleg et al conducted a large retrospective cohort study using databases to evaluate the association of preoperative evaluation with outcomes among patients undergoing noncardiac surgery.^12^ They found a small but statistically significant increase in 30-day mortality, 1-year mortality, mechanical ventilation, and 30-day emergency department visits in the consultation group.^13^ This contrasting evidence suggests that future studies should employ more robust designs focused specifically on patient-centered outcomes. Most of these consultations in our study cohort were requested for low-risk surgical procedures (47%). However, as per literature, utility of consultation service should be prioritized for intermediate risk and high-risk patients^14^ following a coordinated and well-defined process. The process must be adaptable to demographic variations and disease pattern. Over half of such consultations in our study were generated for patients with HTN (68.7%) and DM (68.6%), reflecting the most common comorbid conditions in our region as also reported by the WHO.^15^ Moreover, IHD was the primary reason for majority of cancellations in our patients Similarly, cardiology opinion was requested for 63% of patients, reflecting its critical role in perioperative risk assessment. Given the high prevalence of modifiable risk factors and issues with patient compliance in our setting, these consultations provide a vital opportunity to individualize surgical risk and facilitate pre-operative optimization. This is more important for health care in low resource settings^16^ where such planning can enhance preparedness, mitigate last minute cancellations and hence the cost. 17


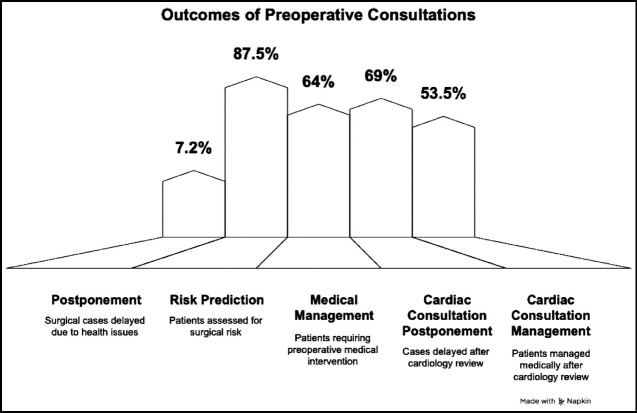


Infographic Abstract.

Streamlining the consultation process is imperative, as literature reports both under- and over-utilization. The cost benefit ratio of such consultations requires critical evaluation^18^, for related concerns of unnecessary testing, adding financial burdens, procedural delays, and unnecessary pre-operative visits.^19^ This is especially crucial in low resource settings and for low-risk surgeries, which accounted for most consultations in our study. Evidence also suggests the total cost of consultations can even exceed the cost of basic diagnostics.^20^ That’s why it require critical insight from the patient point of view.

We have observed similar trends in our daily clinical practice. As these consultations contributes to a substantial delay in clinical care, extend waiting times, and may result in patients for leaving against medical advice. Institutional policies and departmental guidelines need to be formulated to overcome these issues.^21^ Furthermore, the utility of these expert opinions are often influenced by medico-legal considerations, which vary significantly based on a patient’s socioeconomic status and whether their care is delivered in the public or private sector.

To streamline this process, communication between the teams needs to be strengthened. Consultation requests should specify precise questions, with recommendations based on surgical risk and patient-specific factors. Implementing a closed-loop communication system is essential to ensure recommendations are being followed promptly. Furthermore, a shared understanding between surgical and anaesthesia teams regarding cancellations is crucial^22^ for misidentifying the consultations, as the primary cause of cancellations. Nonetheless, utility of these opinions is often necessary in our health care setup for all the forementioned reasons.

### Limitations:

There are certain limitations which are associated with our work. The utility of these consultations needs to be evaluated based on actual outcomes. This includes short term and long-term mortality including the impact on perioperative complications. This requires better study design and long-term follow-up.^23^

## CONCLUSION

The baseline health condition and disease control in our patient population justified the need for preoperative consultations. HTN, DM and IHD were the predominant medical conditions. The outcome of these consultations was mainly risk prediction and optimization. In only 7.2% of patient’s surgical procedure was postponed. Cardiology consultation for patients with IHD is the main reason of high-risk prediction and case postponement.
